# Configurational paths to promoting physical activity among older adults with type 2 diabetes in China: an fsQCA study based on the multi-theory model

**DOI:** 10.3389/fendo.2025.1673625

**Published:** 2025-12-17

**Authors:** Bo Zhang, Panpan Huai, Jintao Wang, Linghui Zhang, Jinli Guo

**Affiliations:** 1 School of Nursing, Shanxi Medical University, Shanxi Province, Taiyuan, China; 2 School of Management, Shanxi Medical University, Shanxi Province, Taiyuan, China; 3 Second Hospital of Shanxi Medical University, Taiyuan, Shanxi, China

**Keywords:** multi-theory model, type 2 diabetes, older adults, physical activity, qualitative comparative analysis

## Abstract

**Objective:**

To deeply analyzes the configurational paths to promoting physical activity in older adults with type 2 diabetes (T2DM), revealing the impact of different combinations of various factors on physical activity.

**Methods:**

This study applied fuzzy-set qualitative comparative analysis (fsQCA) method to identify combinations of Multi-theory Model (MTM) constructs associated with higher physical activity levels.

**Results:**

A total of 1119 older adults with T2DM were included. A single influencing factor does not constitute a necessary condition for promoting physical activity in older adults with T2DM. In contrast, two combinations of the seven influencing factors led to high level of physical activity, and substitutability and complementarity were observed among the various factors of the configuration path.

**Conclusion:**

This study confirmed that the formation of physical activity in older adults with T2DM has multiple concurrent causal relationships and multiple configuration paths. By revealing the synergy among factors, the MTM framework has been expanded. This study offers a novel configuration perspective for understanding the physical activity behavior of this population and provides key evidence for designing diverse physical activity management measures.

## 1 Introduction

Diabetes is a serious public health problem worldwide, with its prevalence rate increasing year by year (1). China, as one of the countries with the largest population in the world, has 148 million people suffering from diabetes by 2024, with a prevalence rate of approximately 10.5%, according to data from the IDF Diabetes Atlas 11th edition (2). With the intensification of global aging, the prevalence of diabetes among the older adults has significantly increased (3). According to the 2024 edition of *the Guideline for the Management of Diabetes Mellitus in the Elderly in China*, there are about 35.5 million older adults in China who have diabetes. This population ranks first globally and accounts for 25% of all older adults with diabetes worldwide. Ninety-five percent of them are type 2 diabetes (T2DM) (4). T2DM is mainly managed comprehensively through lifestyle intervention and individualized drug therapy to achieve blood sugar control. Lifestyle is the main cause of T2DM and can be regarded as the primary preventive measure for its occurrence and development. A study shows that lifestyle intervention can significantly reduce the incidence of T2DM by 58%, compared to a 31% reduction after taking metformin (5–8). Physical activity plays a significant role in the comprehensive management of patients with T2DM and is one of the main forms of lifestyle intervention (9). Individuals within the Finnish Diabetes Prevention Study who reported an increase in their exercise levels were 63–65% less likely to develop T2DM. This suggests a significant physical activity contribution for T2DM prevention and management (8, 10). Physical activity can significantly lower blood sugar levels, prevent and delay complications, relieve anxiety and depression, reduce the cardio-vascular risk, and improve mental well-being and quality of life (9, 11).The impact of physical activity on glycemia and cardiovascular risk is highly significant—paradoxically, the COVID-19 pandemic highlighted this even more, as having more time led many people to pay greater attention to physical activity (due to remote work) and to healthier nutrition (as a result of cooking at home). In this context, it was a very important period of learning for us (12). However, the physical activity status of people with T2DM is not optimistic. The rate of insufficient physical activity among people with T2DM in China is 22.3% (13). In addition, approximately 51.4% of older adults with T2DM in China have suboptimal physical activity levels (14). Compared to the United States (31%) (15) and the global average (45%) (16), the physical activity situation among older adults with T2DM in China is more severe. Thus, it is crucial to investigate the factors that influence physical activity in older adults with T2DM in China.

Influencing the physical activity of older adults with T2DM is a dynamic process involving the interaction of multiple systems and is coordinated and regulated by various factors. There are many studies exploring influencing factors of physical activity. However, the results of qualitative interviews often rely on the subjective interpretation of researchers and cannot quantify the influence weights of different factor combinations. Quantitative research methods such as regression and structural equation models mainly focus on the linear relationships among variables, emphasize overall patterns and lack the diversity of causal pathways. They fail to take into account the diversity, nonlinearity and dynamics of human behavior, neglects the mutual influence among internal factors within different dimensions as well as the interaction between internal and external environments, and is unable to explore the interaction among various influencing factors or consider the impact of multiple factor combinations on the outcome. Qualitative Comparative Analysis (QCA), proposed by Ragin (17) in 1987, is a research method that combines qualitative and quantitative aspects for cross-case comparative analysis based on Boolean algebra and set theory. The QCA, from a holistic perspective, emphasizes the interdependent relationships among conditions and focuses on “configuration effects” rather than the “net effects” of individual variables. Its advantage lies in the ability to identify the relationships among different combinations of condition variables under the same outcome, and answer the necessity and sufficiency of each condition (18). At the same time, Ragin pointed out (19) that in configuration analysis, research variables (conditions or results) must be selected based on theory, which is the deductive aspect of the QCA method.

Physical activity is a long-term process of behavior change. It is not only necessary to promote the initiation of physical activity but also to persist in and maintain it in order to truly exert its positive effects on health. In 2015, Manoj Sharma introduced the Multi-theory Model (MTM), a systematic and integrated theory of health behavior (20). With great accuracy and predictive power, this theoretical model, based on dynamic changes, can be applied at the individual, group, and community levels. Sharma noted that there are two stages to changing one’s health behavior: one is the initiation of health behavior change, and the other is the sustenance of health behavior change. Meanwhile, Sharma pointed out that the influencing factors for initiating health behavior change are different from those for sustained behavior change, which is the core content of this theory. In addition, previous studies have also found that there is a correlation between the factors of the two stages (21). One of the significant advantages of QCA is that it emphasizes the interdependence among factors, examining whether and how the combination of multiple factors affects the outcome. This is consistent with the perspective in the MTM that behavioral change occurs at different stages and is influenced by a combination of factors, which cannot be explained by regression or structural equation models. Additionally, traditional regression is often based on hypotheses to verify results, while QCA emphasizes propositions as the basis for logical deduction. A proposition is a description of logic and theory, and it serves as a prerequisite for an assumption. Verify the influence of logical combinations to illustrate the changes in the dependent variable. The physical activity of older adults with T2DM is influenced by multiple factors. Therefore, QCA can help explore the impact of various factor combinations on physical activity in research. The combination of multiple factors is more meaningful for understanding the factor expressions at different stages in the MTM and provides an integrated approach for the formulation of intervention strategies. This study is the first to attempt to apply the MTM and QCA method to the physical activity of older adults with T2DM. Based on the MTM, it deeply analyzes the configurational paths to promoting physical activity in older adults with T2DM through QCA, revealing the impact of different combinations of various factors on physical activity. It aims to provide practical guidelines for constructing precise and personalized physical exercise management programs for older adults with T2DM.

## 2 Methods

### 2.1 Study model

**Figure 1:**
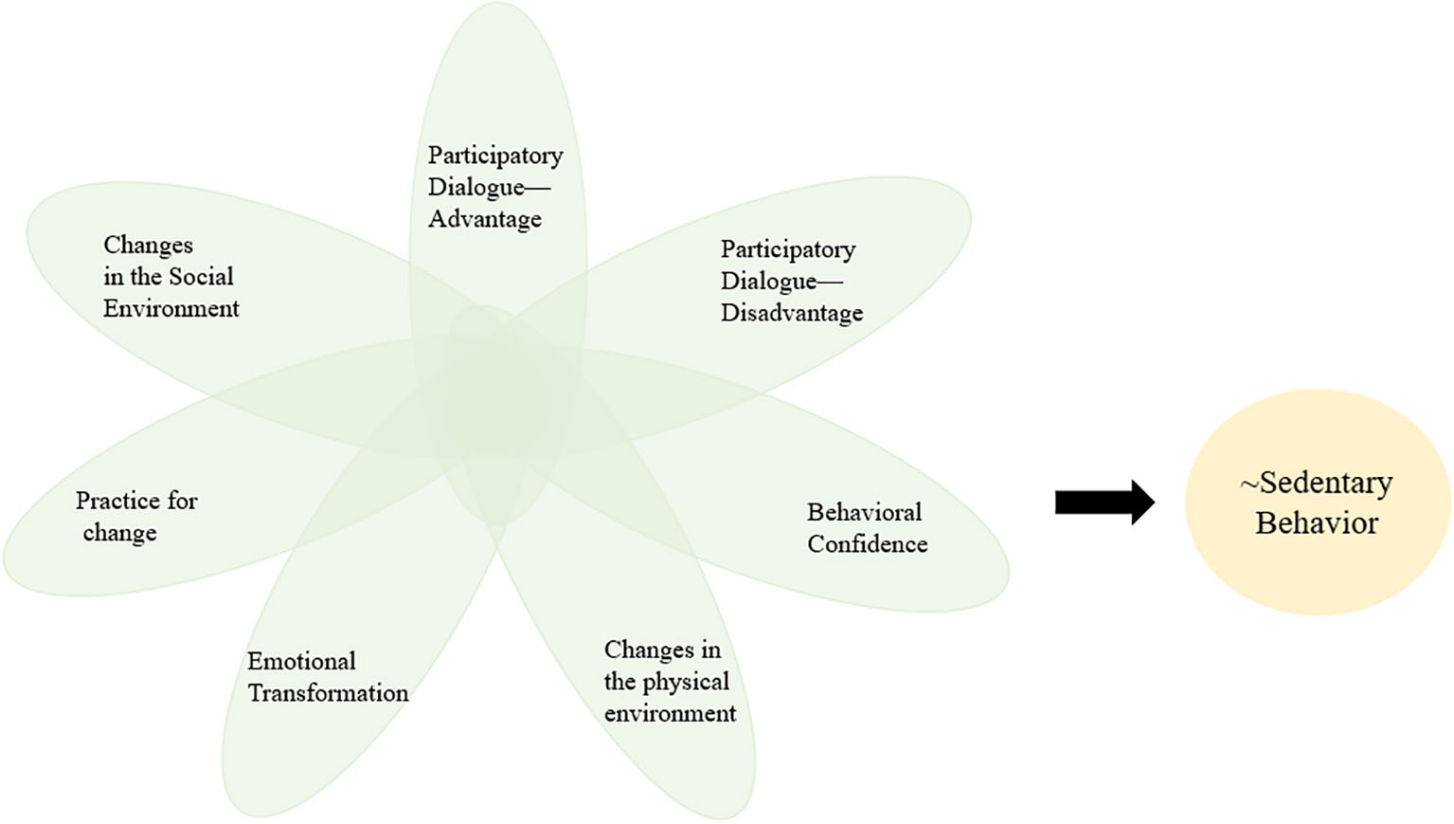
Conceptual model of physical activity.

Based on the MTM, this study hypothesizes that the interaction of factors such as participatory dialogue-advantages, participatory dialogue-disadvantages, behavioral confidence, changes in the physical environment, emotional transformation, practice for change, and changes in the social environment will influence the level of physical activity. This study uses the sedentary behavior to represent the level of physical activity. The World Health Organization often describes and explains physical activity and sedentary behavior together, and pointed out that older adults with chronic conditions should limit the amount of time spent being sedentary. Replacing sedentary time with physical activity of any intensity (including light intensity) provides health benefits (22). Numerous studies have shown that the longer the duration of sedentary behavior, the lower the level of physical activity, they are negative correlation (23). Among them, in a study based upon the National Health and Nutrition Examination Survey (NHANES) dataset, Healy et al. stated that physical activity and sedentary behavior were almost perfectly inversely associated (Spearman’s rho =-0.98) (24). Moreover, the Sedentary Behavior Research Network (SBRN) also highlights this point (25). “~” refers to negation of the condition. Thus, we use “~SB” to represent physical activity. This study proposes the following conceptual model (**Figure 1**): ~SB= f (PDA, PDD, BC, CPE, ET, PC, CSE). The independent variables are summarized in **Table 1**.

**Table 1:** Summary of the variables used in the model.

Variable	Symbol	Indicator
Participatory Dialogue—Advantage	PDA	The main content of participatory dialogue-advantage focused on the advantages of changes in health behaviour.
Participatory Dialogue—Disadvantage	PDD	The main content of participatory dialogue-disadvantage focused on the disadvantages of changes in health behaviour.
Behavioral Confidence	BC	Behavioral confidence highlights a person’s self-assurance in their capacity to form good behavioral patterns.
Changes in the physical environment	CPE	Changes in the physical environment emphasize the importance of individuals’ physical environment, including the availability, accessibility, and convenience of resources, which could also affect behavioral intentions.
Emotional Transformation	ET	Emotional Transformation involves converting or transforming negative and fostering positive emotions toward the health behavior change.
Practice for change	PC	Practice for change entails constantly thinking about the health behavior change and making midcourse corrections to one’s strategy, overcoming barriers, and remaining focused on health behavior change.
Changes in the Social Environment	CSE	Changes in the Social Environment entails creating social support from the environment. This change in the social environment can be natural or artificial.
Sedentary Behaviour	SB	Sedentary behaviour is any period of low-energy expenditure while awake such as sitting, reclining or lying.

### 2.2 Participants

This study conducted the convenient sampling survey from March 2024 to June 2024. The participants were older adults with T2DM in Shanxi Province. To reduce the deviation of the results and ensure the integrity and authenticity of the data, all researchers were pre-trained, conducted the investigation using uniform expressions and maintaining a neutral attitude. Before conducting the investigation, it is necessary to obtain the informed consent of the participants and have them sign the informed consent form. All information and data will be kept confidential. Participants have the right to withdraw at any stage of the research process. Participants were screened according to the inclusion and exclusion criteria.

The inclusion criteria for participants were as follows: (1) Accord with the diagnostic criteria for T2DM as stipulated in *the Guideline for the Management of Diabetes Mellitus in the Elderly in China (2024 edition) (*26) (Panpan Huai and Jinli Guo, experts in endocrinology, conducted the screening based on the diagnostic criteria of the guidelines and in combination with the medical records of the participants);(2) aged sixty years or older;(3) Clear consciousness, no intellectual disability;(4) Obtain the informed consent of the patient/patient and their family. Meanwhile, the exclusion criteria included: (1) Combined with other serious diseases, such as malignant tumors;(2) Having impairments in language communication, hearing and vision;(3) Unwillingness to participate in the investigation;(4) Unclear consciousness;(5) Having significant cognitive deficits or mental disorders;(6) Participating in other research programs.

The researchers will conduct investigation with the patients using the general information questionnaire, sedentary behavior measures and the Measuring Change in Physical Activity Questionnaire. For those research subjects who are capable of filling out the questionnaire, ask them to do it themselves. For patients who were unable to fill out the questionnaire, the investigators read each item of the questionnaire one by one and asked them to answer according to their own situations. During this process, the investigators could not give any hints to the patients and used uniform guidance and instructions to all the participants. Check on the spot whether the patients have filled out the scale completely. If there are any omissions, promptly make up for them to ensure the authenticity and reliability of the scale data information of the research participants. Meanwhile, a random inspection and verification of the questionnaire data was conducted. During the data processing stage, relevant training was provided for data entry and cleaning data work. A parallel double entry method was adopted to ensure the accuracy of data entry. Data entry quality was verified through random checks. Data cleaning involves identifying and filling in missing information, correcting logical errors, and eliminating invalid data.

In the research exploring the influencing factors of relevant variables, the sample size should be at least 5 to 10 times the number of variables (27). According to this research, there were 7 independent variables. Considering that 20% of the samples are inefficient, the calculation requires a sample size of 42 to 84 cases.

### 2.3 Data collection

#### 2.3.1 The general information questionnaire

The general information questionnaire was compiled by the research team based on literature review and data collection, including basic information such as age, gender, residence, marital status, educational level, capita family monthly income, duration of diabetes, treatment regimen, complications, and comorbidities.

#### 2.3.2 Measuring change in physical activity questionnaire

The Measuring Change in Physical Activity Questionnaire (MCPAQ) developed by Professor Sharma (28) et al. is based on a MTM, which includes two sub-scales: initiation of behavior change (MCPAQ-INIT, init=initiation) and sustenance of behavior change (MCPAQ-SUST, sust=sustenance). This scale adopts the Likert 5-point scoring method. This scale was initially developed to assess the physical activity of college students. It also shows good reliability and validity when applied to evaluate the physical activity of other groups. Moreover, this scale can be applied at the individual, group and community levels. In 2019, Yang et al. (29), after obtaining permission from the original authors and completing a cross-cultural adaptation, created a Chinese version of the MCPAQ and validated it among hypertensive patients, demonstrating strong reliability and validity. The overall scale’s Cronbach’s alpha was 0.911, The subscales’ Cronbach’s alpha were 0.813 and 0.903. This Chinese version of the MCPAQ is applicable to patients with chronic diseases.

#### 2.3.3 Sedentary behavior measures

The sedentary behavior measures method in the study by Teychenne et al. (30) was adopted, and the sedentary behavior was evaluated in the form of self-reports. The research subjects reported the sitting time they spent on commuting, going to work, watching TV/playing computer/playing mobile phone and other rest time (reading newspapers/books, playing cards, playing chess) on weekdays and the sitting time they spent on the above activity on weekends. Through the calculation formula: daily sedentary time = (sitting time on weekdays ×5+ Sitting time on weekends ×2)/7 (31).

### 2.4 Method of analysis

FsQCA can not only process small samples but also medium and large samples of data. Moreover, this method can fully reflect the degree of change of sample variables, enhance the dynamic analysis performance of static data samples, and can meticulously observe the impact brought by minor changes in conditions at different levels or degrees. Therefore, fsQCA is the most suitable choice, which can achieve detailed analysis of the gradations in conditions and outcomes. For more detailed information, please refer to the supplementary materials. The analytic approach of fsQCA includes: (1) Data calibration: To convert variables from their original value to a fuzzy membership between 0 and 1. Three threshold points were set in this study: the crossover point, the full nonmembership, and the full membership (32). Based on prior empirical research (19, 33), we used the variables’ median value, lower quartile value, and upper quartile value and calibrated the data through the Calibrate function. **Table 2** displays the calibration values for these variables. (2) Necessity analysis: We conducted necessity analysis of all dependent variables and their negations (34). (3) Truth table construction and refinement: Based on the variables’ transformed fuzzy-set membership scores, a truth table was created. The truth table was refined by setting the frequency cutoff at six and the consistency threshold at 0.8, as suggested by Ragin (19). (4) Configuration analysis: This study conducted a configuration analysis for the presence of a high level of physical activity using the fsQCA software. This step also distinguished core conditions and periphery conditions in each solution. (5) Predictive validity analysis: The ability of the hypothesis configuration model to forecast the outcome variable under various data sets was confirmed using predictive validity analysis (35, 36). (6) Sensitivity analysis: The robustness of the results was further examined through sensitivity analysis using alternative condition specifications (37). (7) *Post hoc* analysis: The Tobit regression model is a type of regression model used to process situations where the dependent variable is censored or truncated (38). Because fuzzy set data has upper and lower limits, Tobit regression is more suitable for analyzing such data than other methods, avoiding result deviations (39). Moreover, fsQCA is based on logical analysis of conditional combinations, and through Tobit regression, the relative importance and explanatory power of paths can be evaluated statistically (40).

**Table 2:** Descriptive statistics for dependent and independent variables.

Main descriptions	PDA	PDD	BC	CPE	ET	PC	CSE	SB
Mean	11.71	10.13	10.97	6.11	6.04	5.95	6.75	5.69
SD	5.29	4.80	5.25	3.03	3.03	3.08	3.57	1.63
Minimum	0	0	0	0	0	0	0	0
Maximum	20	20	20	12	12	12	12	8
Percentile								
25	9	7	8	4	4	4	4	5
50	12	10	11	6	6	6	7	6
75	15	13	15	8	8	8	9	7

### 2.5 Ethical approval

This study was approved by the Ethics Committee of the Second Hospital of Shanxi Medical University(approval number:2023YX288). All of the participants or their legal guardians gave their informed consent to participate.

## 3 Results

### 3.1 Sociodemographic characteristics

Ultimately, 1119 older adults with T2DM were invited to participate in this study. The participants’ social demographic characteristics are shown in **Table 3**.

**Table 3:** Descriptive statistical analysis of sociodemographic characteristics.

Sociodemographic characteristics	n (%)
Gender	
Male	537 (47.99%)
Female	582 (52.01%)
Age (years)	
≥60,<70	747 (66.76%)
≥70,<80	282 (25.20%)
≥80	90 (8.04%)
Residence	
Urban	775 (69.26%)
Rural	344 (30.74%)
Educational level	
Elementary school and below	165 (14.75%)
Middle school	353 (31.55%)
High school or technical secondary school	331 (29.58%)
College or bachelor degree	216 (19.30%)
Master and above	54 (4.83%)
Marital status	
Single	162 (14.48%)
Married	894 (79.89%)
Divorced	15 (1.34%)
Widowed	48 (4.29%)
Living status	
Living alone	204 (18.23%)
Living with spouse	594 (53.08%)
Living with childern	78 (6.97%)
Living with spouse and childern	243 (21.72%)
Capita family monthly income (yuan)	
<1000	99 (8.85%)
1000-3000	300 (26.81%)
3000-5000	528 (47.18%)
>5000	192 (17.16%)
Duration of diabetes (years)	
≤1	204 (18.23%)
>1,≤5	369 (32.98%)
>5,≤10	282 (25.20%)
>10,≤15	93 (8.31%)
>15	171 (15.28%)
Complication	
Yes	380 (33.96%)
No	739 (66.04%)
Comorbidity	
Yes	460 (41.11%)
No	659 (58.89%)
Treatment regimen	
None	288 (25.74%)
Oral hypoglycemic agent	513 (45.84%)
Insulin	75 (6.70%)
Oral hypoglycemic agent + insulin	195 (17.43%)
Others	48 (4.29%)

### 3.2 Results of the necessary condition analysis

The results of the necessity test with regard to the impact of each antecedent variable on the outcome variable are shown in **Table 4**; Because physical activity and sedentary behavior were relatively strong inversely associated, we use “~SB” to represent physical activity. The consistency value of each condition and its negation are all below 0.9, thus indicating that no necessary condition for the outcome variable is included among the antecedent variables in this study and that it is difficult for a single antecedent variable to act directly on the outcome variable. It is indicated that the level of physical activity is not caused by a single condition but is the result of the interaction of each condition. Further exploration of the influence of combinations of conditions on physical activity is needed.

**Table 4:** Necessity testing results.

Independent variable	Dependent variable: ~SB	
	Consistency	Coverage
PDA	0.85	0.79
~PDA	0.26	0.25
PDD	0.80	0.73
~PDD	0.32	0.31
BC	0.83	0.79
~BC	0.31	0.29
CPE	0.82	0.76
~CPE	0.31	0.30
ET	0.82	0.76
~ET	0.29	0.28
PC	0.80	0.75
~PC	0.33	0.31
CSE	0.85	0.77
~CSE	0.26	0.26

“~” refers to negation of the condition.

### 3.3 Results of the configuration analysis

**Figure 2:**
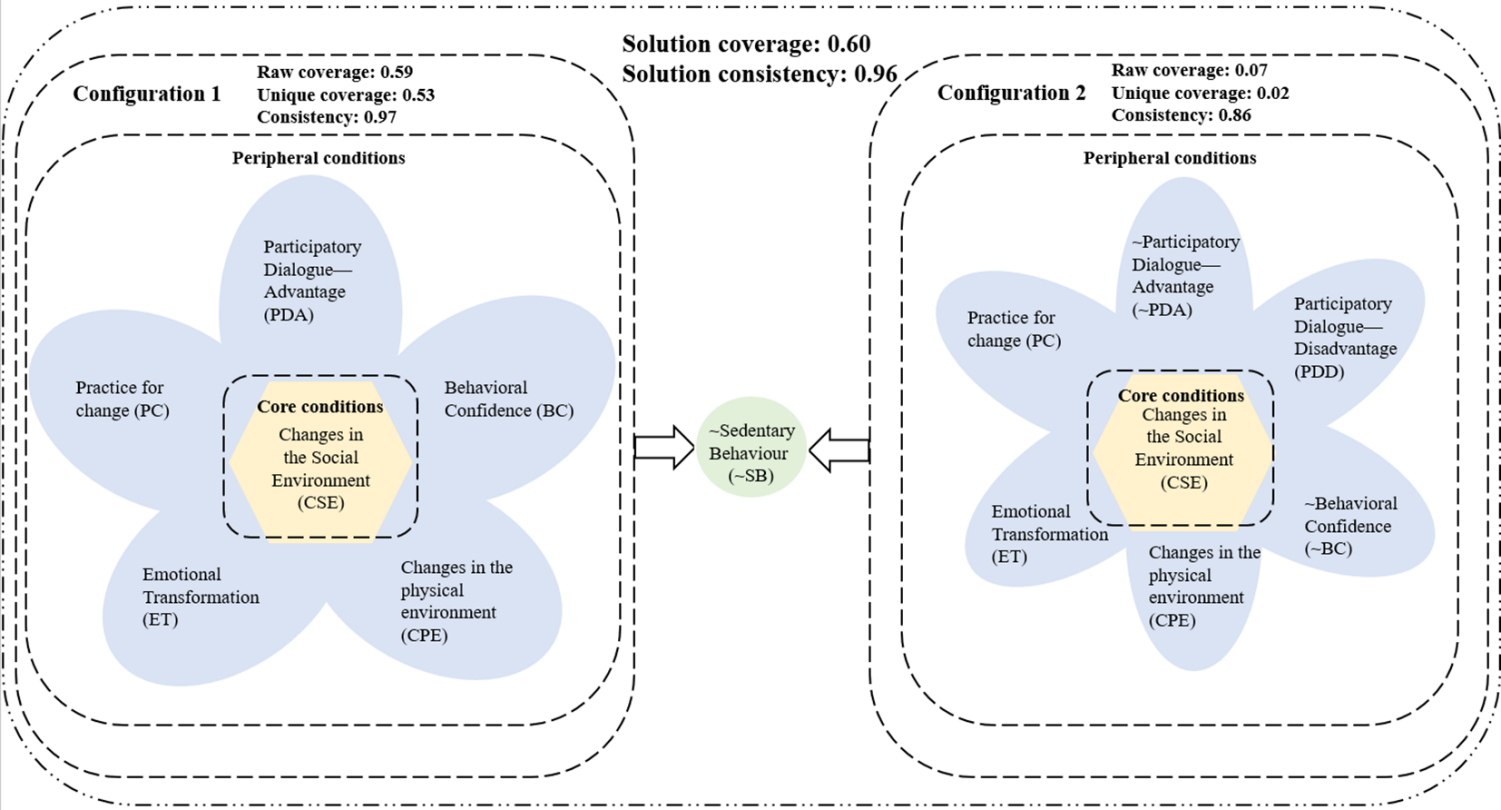
The presentation of configuration analysis results. “~” refers to negation of the condition.

The solutions of the configuration analysis are shown in **Tables 5**, **6**, **Figure 2**. The configuration’s parsimonious solution for a high level of physical activity only offers one path. While factors that appear in complex solutions but do not appear in related parsimonious solutions are periphery conditions leading to the outcome, those that appear in parsimonious solutions are core conditions leading to the outcome. Core attributes indicated a strong causal relationship between the antecedent condition and the outcome, while peripheral attributes indicated a weaker causal relationship, which is a condition that plays an auxiliary role (18). The core conditions (In this study, it is Changes in the Social Environment (CSE)) served as the basis for the creation of the two intricate solutions for a high degree of physical activity, which we subsequently refer to as Configurations 1 and 2. Overall solution coverage was 0.6 and overall consistency was 0.96. The suggested threshold values of 0.8 and 0.5 were attained by these two values, respectively. The consistency scores of each solution ranged from 0.85 to 0.96, exceeding the recommended threshold values of 0.8. These indicate that the data adjusted well to all factors combinations. Our detailed interpretation of the two solutions is enumerated below.

**Table 5:** Results of the configuration analysis.

Solutions	~SB
Parsimonious solution	CSE
Complex (intermediate) solution	PDA*BC*CPE*ET*PC*CSE
	~PDA*PDD*~BC*CPE*ET*PC*CSE

“*” means Boolean logic “and”; “~” refers to negation of the condition.

**Table 6:** Configurations for achieving physical activity.

Factor	~SB	
	Configuration 1	Configuration 2
Participatory Dialogue—Advantage	•	⊙
Participatory Dialogue—Disadvantage		•
Behavioral Confidence	•	⊙
Changes in the physical environment	•	•
Emotional Transformation	•	•
Practice for change	•	•
Changes in the Social Environment	●	●
Raw coverage	0.59	0.07
Unique coverage	0.53	0.02
Consistency	0.97	0.86
Solution coverage	0.60	
Solution consistency	0.96	

•indicates the presence of a condition and ⊙indicates its absence. Large circles indicate core conditions. Small circles indicate peripheral conditions. Blank spaces indicate that the condition is indifferent.

1. Configuration 1 identified the one core condition as high levels of changes in the social environment, together with five periphery conditions including high levels of participatory dialogue—advantage, behavioral confidence, changes in the physical environment, emotional transformation and practice for change.
2. Configuration 2 identified one core condition consisting of high levels of changes in the social environment, together with six periphery conditions including low levels of participatory dialogue—advantage, high levels of participatory dialogue—disadvantage, low levels of behavioral confidence, high levels of changes in the physical environment, high levels of emotional transformation and high levels of practice for change.

### 3.4 Results of predictive validity

**Figure 3:**
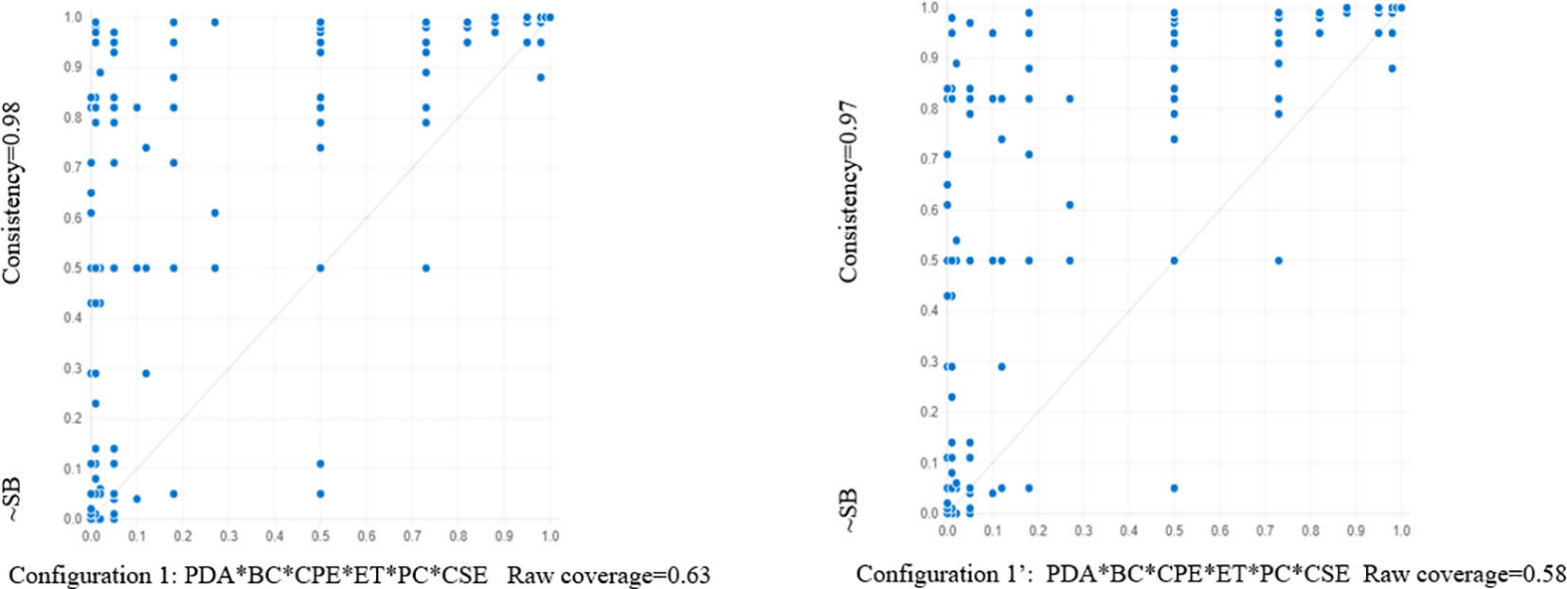
XY scatterplots of physical activity in configurations 1 and 1’. Left: Configuration l (by Sub-sample l), Right: Configuration l’(by Sub-sample 2).

Random selection was used to divide the original sample into two equal sub-samples: a modeling sub-sample (Sub-sample 1) and a holdout sample (Sub-sample 2). Using the same frequency cutoff and consistency threshold as the main analysis, fsQCA was carried out for Sub-sample 1 (41). Later, data from Sub-sample 2 was used to test the configurations in Sub-sample 1. Similar consistency and coverage of results were attained by all model tests (**Figure 3** shows the model testing of Configuration 1). As a result, the suggested configurations demonstrated strong predictive power across various data sets.

### 3.5 Results of sensitivity analysis

We describe the operations and results of the sensitivity analysis in detail, which involved altering the anchor systems, frequency cutoff, and consistency threshold to carry out the robustness tests (37). (1) the three points (full membership, full nonmembership, cross-over point) of calibration in this study were the 75th, 25th, and median, respectively. We changed these to the 80th, 20th, and median, respectively, to conduct the first sensitivity analysis; (2) The case frequency cutoff in this study was “6”. We changed this from “6” to “9” to conduct the second sensitivity analysis. This means that only a row in the truth table with at least 9 cases could be included in the configuration analysis; (3) The consistency threshold of this study was 0.8. We changed it from “0.8” to “0.85” to conduct the third sensitivity analysis. This change means only the rows in the truth table with a consistency score over 0.85 could be included in the configuration analysis.

The detailed results of the assessment indices are displayed in **Table 7**. The sensitivity analysis results indicated that, despite minor changes to the neutral permutations that occur and the specific number of solutions and sub-solutions, the interpretation of the results remained substantively unchanged.

**Table 7:** Results of sensitivity analysis.

Configurations	Changed thresholds form 75^th^, 50^th^, 25^th^ to 80^th^, 50^th^, 20^th^	Changed case frequency cutoff from at least 6 to 9 cases	Changed the consistency threshold from 0.8 to 0.85
Configuration 1: PDA*BC*CPE*ET*PC*CSE	✔	✔	✔
Configuration 2: ~PDA*PDD*~BC*CPE*ET*PC*CSE			✔
Solution coverage	0.61	0.59	0.60
Solution consistency	0.97	0.97	0.96

✔means the solution exists; blank spaces mean the solution does not exist.

### 3.6 Results of *post hoc* analysis

Both Configuration 1 and Configuration 2 had a positively significant effect on physical activity (*β* = 1.42; *P*<0.01), according to the *post hoc* analysis results (**Table 8**). These outcomes roughly match the ones that fsQCA produced.

**Table 8:** Results of the Tobit regression analysis for physical activity.

Independent variable	Coefficient (*β*)	Standard error	z-statistic	Probability
Dependent variable: ~SB				
Configuration 1	1.42	0.03	44.35	<0.01
Configuration 2	0.96	0.12	7.87	<0.01

## 4 Discussion

Based on the MTM, the results of fsQCA present two configuration paths, and each path is composed of multiple factors. This indicates that the generation of physical activity is jointly determined by multiple influencing factors and is the result of the mutual influence and synergy of multiple factors. The diverse paths presented among the configurations reflect that older adults with T2DM have different needs and perceptions of physical activity (42), and there are multiple pathways or multiple causal relationships for the improvement of their physical activity levels. Therefore, the improvement of physical activity levels in older adults with T2DM not only requires considering the influence of multi-dimensional internal factors, but also needs to establish diversified physical activity management plans and implementation strategies based on the multiple combined effects of each influencing factor (42).

### 4.1 Core condition

Changes in the social environment entails creating social support from the environment. This change in the social environment can be natural or artificial. Changes in the social environment stem from concepts such as environmental structure, helping relationships, and social support (43, 44). Social support mainly comes from friends, family and medical professionals, and occasionally from the patients themselves. Emotional, instrumental, informational, and appraisal are the four categories into which a widely used definition of social support divided the functional content of relationships (45). Emotional support encompasses actions that increase our sense of self-worth and make us feel loved and cared for. Such assistance may take the form of encouragement or discussing an issue. Conversely, instrumental support encompasses the different forms of material assistance that are given by others, such as sports watches and glucometers. Third, informational support refers to assistance that others may provide in the form of information, suggestions, or advice. Affirmation, feedback, social comparison, and information for self-evaluation are the final components of appraisal support. Some studies suggest that social support can affect engagement in T2DM management by influencing emotions, cognitions, and behaviors (46), and it can also directly influence such health-related behaviors such as physical activity, diet, smoking, sleep, and adherence to medical regimens. When analyzing the continuous impact of social environment changes on physical activity through our previous qualitative interviews, it was found that patients mentioned major support from patients, peers and family members. Whereas, as an essential part of the social environment change-professionals, like community health education nurses and health education experts, are rarely mentioned, which are an important force in the changing social environment and can provide professional, precise and targeted health education and management solutions. Meanwhile, COLL et al. also hold that social support from healthcare providers is a necessary condition for promoting patients’ physical activity levels (47). The management of diabetes focuses on the community. Compared with community health systems of other countries, China’s community health services started late, developed immaturely, and the service quality is uneven (48). The human resources of community health service are insufficient, and the ratio of community health service providers to community residents is unbalanced, resulting in the community health service providers cannot take into account the health status of each community patient. In addition, community health service providers lack professional medical treatment and nursing service, and their specialty literacy is generally low (49). To solve this problem, it is possible to invite some teachers from medical colleges to provide regular training and assessment for community health service providers, thereby enhancing the overall quality of community health service providers. Additionally, experts from hospitals can be invited to offer regular free clinic to reduce the medical expenses for residents and provide more convenient medical services (21).

### 4.2 Periphery conditions

The main content of participatory dialogue focused on the advantages and disadvantage of changes in health behavior. However, numerous studies have shown that the older adults’ understanding of physical exercise only remains at the basic function of strengthening the body and keeping healthy. They have not truly recognized the preventive and control role of “Sports-Medicine Integration” in chronic diseases, especially the role of physical exercise in regulating blood sugar (50). Moreover, due to frequent relapses and increasing complications, older adults with T2DM often lack confidence and feel anxious, making them feel that they cannot control the condition or change the current situation (51). This leads to severe psychological burdens and even neglecting blood sugar management. Therefore, the medical staff should help patients understand the basic knowledge of diabetes and physical activity, promptly identify patients’ negative emotions, eliminate inappropriate predictions, misunderstandings, and wrong beliefs, enhance their confidence in curing the disease, and assist them in adhering to medication, regular physical activity, and formulating dietary control plans, etc. In China, older adults with T2DM mostly live at home and in the community, where they carry out long-term self-management and receive community care. However, currently, there are relatively few professional fitness facilities suitable for older adults in the community, and some facilities are outdated, having potential safety hazard. It is urgent for the government departments to carry out aging-friendly renovations of community facilities and provide safety guarantees (52). Practice for change entails constantly thinking about the health behavior change and making midcourse corrections to one’s strategy, overcoming barriers, and remaining focused on health behavior change (53). According to the results of a mixed-methods study, many older adults with T2DM will adjust their physical activity levels based on their dietary conditions and blood sugar levels (42). In addition, internal factors such as diseases, financial situation, BMI, age, lack of time, fatigue, lack of a companion or motivation, and education level can affect the level of physical activity (8, 54–56). External factors also affect patients’ physical activity, such as the prevalence of infectious diseases, bad weather, air quality, interpersonal relationships, work and family responsibilities, etc (57). Therefore, it is worth considering timely adjustment of physical exercise programs and choosing alternative and flexible physical exercise programs. With the increasing maturity of big data technology, user profiles and information platforms have been widely applied in the field of precise and personalized services. In the future, combining user profiles and information platforms with physical exercise programs for older adults with T2DM will help improve the accuracy of physical exercise data for older adults with T2DM, promote data resource sharing among sports and medical and health platforms, effectively monitor and evaluate the implementation of physical exercise programs for older adults with T2DM, and achieve precise and effective supply of physical exercise programs for older adults with T2DM, providing new ideas for designing personalized physical exercise programs for older adults with T2DM (58).

### 4.3 Configuration paths

Interestingly, although the physical activity of older adults with T2DM is the result of the interaction of multiple variables, the explanatory power of the results for promoting physical activity varies under different conditions. From the two configuration paths, it can be seen that changes in the social environment exist in both configuration paths and serve as the core condition. This suggests that this influencing factor has an important degree and core position in promoting physical activity for healthcare professionals. At the same time, this study found that participatory dialogue—advantage and behavioral confidence did not show a positive effect in both paths as expected, and participatory dialogue—disadvantage did not show a negative effect. Instead, it was more dependent on the synergy and mutual influence produced by the combination with other variables, and worked together with other factors as periphery conditions to act upon the core condition. When these influencing factors are combined together, they play a relatively important role in generating the promotion of physical activity.

### 4.4 Limitations

There are multiple limitations to this study. First off, there was bound to be subjective bias because the data used in this study came from a questionnaire survey. Secondly, there were not many conditional variables examined in this study, only including the relevant variables related to the MTM. Additional factors that influence the path of physical activity should be considered in subsequent research. Thirdly, the samples collected in this study are all from Shanxi Province, China. In the future, the sample size should be increased and groups from different regions should be included to ensure the comprehensiveness and representativeness of the research results. Finally, in this study, sedentary behavior was used as a substitute for physical activity. Although there was a relatively strong negative correlation between the two, they could not completely replace each other. Therefore, in the future, more scientific and effective measurement tools for physical activity should be used to make the article more standardized and rigorous.

## 5 Conclusion

Based on the MTM, this study, for the first time, used the fsQCA method to take 1119 older adults with T2DM in Shanxi Province, China as the research subjects to explore the configuration paths influencing the physical activity of older adults with T2DM. This study explored two configuration paths with different combinations of influencing factors and conducted interpretations. This suggests that when formulating physical activity intervention measures, medical staff should take into account that the management of physical activity is diverse and that physical activity levels can be enhanced through multiple paths. It is not sufficient to merely consider a single factor or causal relationship. And combined with the different roles of each influencing factor in the configuration to adopt corresponding management measures, which is more conducive to improving the physical activity status of older adults with T2DM.

## Data Availability

The original contributions presented in the study are included in the article/supplementary material. Further inquiries can be directed to the corresponding authors.
